# Differential regulation of motility and immune synapses by CD28/ CTLA-4 costimulation in effector and regulatory T cells

**DOI:** 10.1186/1479-5876-10-S3-P6

**Published:** 2012-11-28

**Authors:** Nahzli Dilek, Nicolas Poirier, Philippe Hulin, Gilles Blancho, Bernard Vanhove

**Affiliations:** 1 Institute of Transplantation Urology Nephrology, University of Nantes, INSERM UMR 1064, Nantes, France Effimune, Nantes, France; 2 IMPACT INSERM platform, Nantes, France

## Background

We have previously shown that antagonist anti-CD28 antibodies block CD28/CD80-86 costimulation without perturbation of the CTLA-4/CD80-86 inhibitory pathway and favor tolerance induction by increasing Treg suppression in a CTLA-4 dependent manner. Since CTLA-4 is transducing signals that block the TCR-STOP signal, described to allow for T cell arrest and formation of immune synapses, we hypothesized that CTLA-4 might play a major role in the mechanism of action of anti-CD28 antibodies by regulating T cell motility and synapses formation.

## Materials, methods and results

Here, we generated human CD4^+^CD25^+^CD127^+^ Teff and CD4^+^CD25^high^CD127^low^Foxp3^+^ Treg cell lines and analyzed their behavior in contact with cognate APCs by live-cell dynamic microscopy in the presence of CD28 and CTLA-4 antagonists. CD28 blockade prevented formation of stable contacts between Teff and APCs (11.93 ± 1.175 vs 4.167 ± 1.191 min; p<0.05), increased Teff mobility (100.5 ± 6.032 *vs* 204.8 ± 17.54 µm; p<0.0001) and decreased cell activation measured by calcium flux (0.377 ± 0.028 vs 0.154 ± 0.024 calcium peaks/min; p<0.0001). In contrast, CD28 antagonists enhanced Treg/APC contacts (5.057 ± 0.866 vs 13.81 ± 1.104 min; p<0.0001) and increased calcium flux (0.486 ± 0.048 vs 0.677 ± 0.06 calcium peaks/min; p<0.05), resulting in an increase of Treg activation. The simultaneous blockade of CTLA-4 with antibodies or of CD80/86 with CTLA4Ig reversed some of these effects: it restored the STOP signal and reduced motility/velocity in Teff whereas it increased velocity in Treg and abolished Treg/APC contacts.

## Conclusion

Our data shed light on the role of CD28 and CTLA-4 that act as a rheostat to differentially control Teff and Treg function and clarify the observations that selective CD28-blockade but not CD80/86 blockade reinforces Treg cell suppression *in vitro*.

